# Anatomical Links between White Matter Hyperintensity and Medial Temporal Atrophy Reveal Impairment of Executive Functions

**DOI:** 10.14336/AD.2018.0929

**Published:** 2019-08-01

**Authors:** Takehiko Yamanaka, Yuto Uchida, Keita Sakurai, Daisuke Kato, Masayuki Mizuno, Toyohiro Sato, Yuta Madokoro, Yuko Kondo, Ayuko Suzuki, Yoshino Ueki, Fumiyasu Ishii, Cesar V Borlongan, Noriyuki Matsukawa

**Affiliations:** ^1^Department of Neurology,; ^2^Department of Radiology,; ^3^Department of Rehabilitation Medicine, Nagoya City University Graduate School of Medical Sciences, Nagoya 467-8601, Japan.; ^4^Department of Health Sciences, Nihon Fukushi University, Higashihaemi-chou, Aichi 475-0012, Japan.; ^5^Department of Neurosurgery and Brain Repair, University of South Florida College of Medicine, Tampa, FL 33612, USA.

**Keywords:** Alzheimer’s disease, executive function, quantitative analysis, white matter hyperintensity

## Abstract

Although several studies have demonstrated correlation between white matter hyperintensities (WMH) and impairment of executive functions, the underlying anatomical-functional relationships are not fully understood. The present study sought to investigate the correlations between the volume of WMH and medial temporal lobe atrophy (MTA) using quantitative magnetic resonance image (MRI) and a variety of executive function assessments. A total of 91 patients ranging in age from 58 to 90 years with mild cognitive impairment (MCI) due to Alzheimer’s disease (AD) or early phase AD were recruited from the outpatient clinic at the Department of Neurology of Nagoya City University Hospital. We administered neuropsychological batteries evaluating verbal memory, orientation, spatial ability, sustained attention, and a variety of executive functions, including verbal fluency, flexibility, inhibition, and working memory. Quantitative MRI analyses were performed using Dr. View/Linux software and a voxel-based specific regional analysis system. Significant correlations were observed between WMH, as well as MTA, and some executive function scores. Regression analysis revealed that MTA was the strongest predictor of flexibility and verbal fluency. These findings provide new insight into the relationship between quantitative MRI analyses and various types of executive dysfunction in elderly people with MCI due to AD and/or early phase AD. When cognitive function is examined in elderly patients with MCI due to AD or early phase AD, it is important to consider the involvement of WMH and MTA, which is indicative of AD pathology in cognitive dysfunction, particularly executive function.

Assessments of subtle structural changes in the white matter using quantitative magnetic resonance imaging (MRI) analysis have provided insight into the mechanisms underlying both normal brain aging and pathological processes [1-5]. White matter hyperintensities (WMH), as shown on T2-weighted imaging (T2WI) or fluid-attenuated inversion recovery (FLAIR) images, are associated with neuronal loss, demyelination, and gliosis on neuropathological examination. These features have also been linked to cerebral hypoperfusion and reduced white matter integrity [6-8].

The degree of cognitive impairment associated with WMH is generally accepted to depend on the volume and topography of the lesions [4, 9]. In addition, WMH have been found to induce deficits in executive function by reducing the functional connectivity of the prefrontal cortex with other (sub-)cortical regions [10]. Studies examining the correlations between WMH and executive functions have adopted verbal fluency, flexibility, inhibition, and working memory as measures of executive function. Although these functions include cognitive processes, not all functions are affected by WMH. In particular, medial temporal lobe atrophy (MTA), which often coexists with WMH in elderly people, may play a crucial role in executive function, as well as memory performance. Functional connectivity exists between the prefrontal cortex and the medial temporal lobe, implying that MTA is involved in the cognitive characteristics of the prefrontal cortex, including executive functions [11, 12].

However, few studies of aging without dementia have assessed MTA when examining the relationship between WMH and executive functions. Furthermore, WMH can be divided into two anatomically distinct regions: (a) WMH in the area adjacent to the ventricles (periventricular hyperintensities; PVH) and (b) WMH in the area under the cortex (deep and subcortical white matter hyperintensities; DSWMH) [2, 13]. Importantly, these forms of WMH have different clinical and pathological features. The main differences include that PVH link to atrophic processes involving ventricular enlargement and DSWMH to ischemic risk factors [14]. The distinctive effects of PVH and DSWMH in cognitive dysfunction are currently not fully understood.

In the present study, we used quantitative MRI analysis to investigate the correlations between the volume of PVH, DSWMH, and MTA, and cognitive function, including a variety of executive functions.

## MATERIALS AND METHODS

### Study participants

Patients with mild cognitive impairment (MCI) due to Alzheimer’s disease (AD) and patients in the early phase of AD were recruited from the outpatient clinic at the Department of Neurology at Nagoya City University Hospital. Because MCI due to AD and early-phase AD comprise a continuum of cognitive decline, both groups were combined for our primary analysis. A total of 91 patients ranging in age from 58 to 90 years were included when they were diagnosed as meeting the Petersen’s criteria for MCI and the criteria of the National Institute on Aging and the Alzheimer’s Association for AD (NIA-AA) [15, 16], and had a Clinical Dementia Rating (CDR) score less than or equal to 1, to confirm early stage AD [17]. Since the educational level effects are well known to be strong, the patients who had a minimum of junior high school degrees were included. Exclusion criteria included contraindications for imaging (e.g., brain surgery, cardiac pacemakers, metal implants, claustrophobia, large body tattoos); major vascular disorders such as stroke, including asymptomatic lacunar stroke that incidentally identified by MR scan, heart disease, or other causes of vascular dementia; psychiatric disorders such as major depression, schizophrenia, bipolar disorder, psychotic disorder not otherwise specified, or treatment for a psychotic disorder with psychotropic drugs, including benzodiazepines, anticonvulsants, and anticholinergics, within the previous 12 months; epilepsy; Parkinson’s disease; multiple sclerosis; electroshock therapy; kidney dialysis; Meniere’s disease; infection, trauma, or major structural abnormalities of the brain; cognitive impairment due to alcohol and/or drug abuse or abuse of other substances; and absence of a reliable person who knows the patient and had detailed knowledge of their concerns. Informed consent was obtained for participation in the experiments. The study was approved by the Institutional Review Board of Nagoya City University Hospital.

### MRI examinations

**Figure 1.**
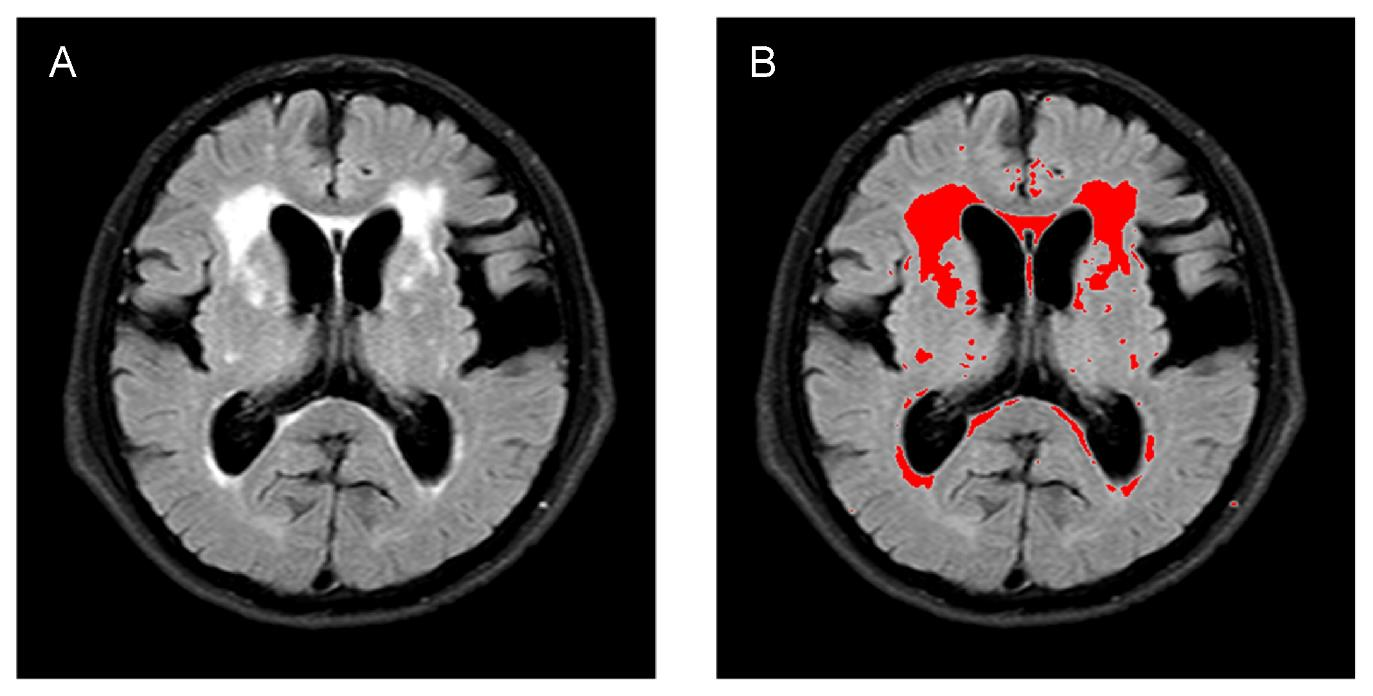
Quantitative analysis of white matter hyperintensity volume A representative fluid-attenuated inversion recovery image (A). Using the bitmap statistics method in Dr. View/Linux, white matter hyperintensity volume segmentations are automatically generated (B).

A 1.5 Tesla scanner was used for brain MRI (Magnetom Vision, Siemens, Germany). A standardized imaging protocol consisting of sagittal T1-weighted (repetition time [TR] 540 ms, echo time [TE] 12.0 ms) and axial T2WI (TR 4000 ms, TE 99.0 ms) and FLAIR (TR 10,000 ms, TE 160 ms) with 5 mm contiguous sections was performed to measure total WMH volume. A T1-weighted 3D sagittal volumetric gradient echo sequence image (TR 9.7 ms, TE 4.0 ms, flip angle 12°, acquisition matrix 256 × 256, 1.2-mm slice thickness) was acquired for voxel-based morphometry (VBM) analysis and hippocampal tracing. For measurement of WMH volume, we used semi-automated quantitative image processing software (Dr. View/Linux, Asahi-Kasei Information Systems, Tokyo, Japan) on a Linux workstation [18]. WMH were defined as hyperintense lesions on every 23 slices from T2WI or FLAIR images. By combining fuzzy clustering, connectivity rules and mathematical morphology, WMH segmentations were automatically generated (Fig. 1). While WMH connected to the lateral ventricles were labelled PVH, WMH not connected to the lateral ventricles were labelled DSWMH. We used information from the white matter parcellation atlas to create regions of interest for DSWMH. Among them, DSWMH of the frontal lobe (DSWMH-F) were featured and entered as independent variables in a step-wise multiple linear regression model. Using the bitmap statistics method in Dr. View/Linux, we calculated the total volume of WMH, PVH, DSWMH, and DSWMH-F. In addition, we used the Voxel-based Specific Regional Analysis System for Alzheimer’s Disease (VSRAD), an automated software program, for the diagnosis of AD using the VBM technique [19], to evaluate MTA. VSRAD enables the evaluation of the degree of entorhinal cortex and parahippocampal volume loss by comparing a given subject’s gray matter volume with that of the original healthy individual database template. By comparisons with the image database for healthy individuals, Z scores (the magnitude of gray matter density’s discrepancy, n × standard deviation [SD], in individual patients from the mean for healthy individuals) reflect the degree of atrophy of the bilateral entorhinal cortex and a Z-score >2 indicates significant atrophy of the hippocampal region. Average Z-scores of left and right MTA were used for the analyses.

### Cognitive assessments

First, patients were screened using the Mini-Mental State Examination (MMSE) [20] and CDR [17], which are also applicable for MCI due to AD or the early stages of AD according to the NIA-AA criteria. Second, all study participants underwent cognitive testing with a comprehensive test battery that covered verbal memory, orientation, spatial ability, sustained attention, and a variety of executive functions, including verbal fluency, flexibility, inhibition, working memory. Briefly, these executive functions were measured using the trail making test (TMT) [21], the completion time of the TMT part B, corrected for part A (TMT-B/A), the digit span test backward (DST-B) [22], the modified Stroop test (MST) [23], semantic verbal fluency (SVF) [24], and the frontal assessment battery (FAB) [25]. For the other domains, we conducted subtests from the Alzheimer’s Disease Assessment Scale - Cognition (ADAS-Cog) [26], including 10 words delayed recall (10WDR), orientation, and visuospatial ability.

### Statistical analysis

Partial correlations between cognitive functions and PVH, DSWMH, DSWMH-F, and MTA were calculated, controlling for age and years of education. In addition, to determine the unique contributions of PVH, DSWMH, DSWMH-F, and MTA to cognitive functions, hierarchical multiple regression analyses with stepwise selection were performed while controlling for age and years of education. The significance level was set at p < 0.05. Statistical analyses were performed using MATLAB software (The Mathworks Inc., Natick, MA).

**Figure 2.**
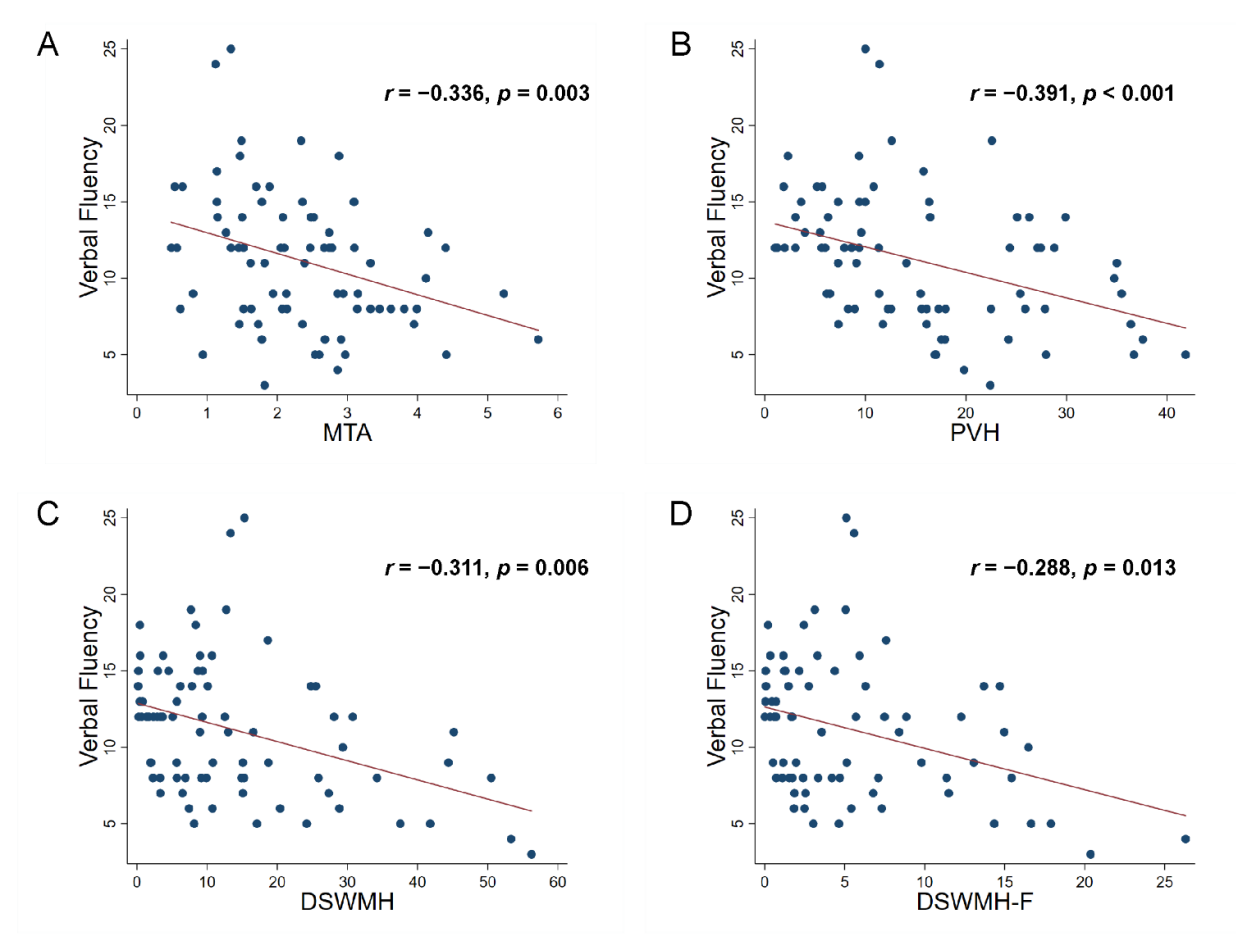
Correlation analyses between verbal fluency scores and imaging parameters Significant negative correlations were confirmed with MTA (r = -0.336, p = 0.003; A) PVH (r = -0.391, p < 0.001; B) DSWMH (r = -0.311, p = 0.006; C) DSWMH-F (r = -0.288, p = 0.013; D) MTA = medial temporal lobe atrophy; PVH = periventricular hyperintensity; DSWMH = deep and subcortical white matter hyperintensity; DSWMH-F = deep and subcortical white matter hyperintensity of frontal lobe.

## RESULTS

### Clinical characteristics

A total of 91 patients ranging in age from 58 to 90 years, with MCI due to AD or early phase of AD were studied. Clinical characteristics of the study participants are presented in Table 1.

### Correlation between cognitive performance and MRI variables

Correlations between cognitive functions, including a variety of executive functions, and the volume of WMH, and Z-scores of MTA, are presented in Table 2. The results revealed strong correlations between MTA and verbal memory (r = -0.309, p < 0.01) and orientation (r = -0.301, p < 0.01). Sustained attention was significantly correlated with PVH (r = 0.367, p < 0.01), DSWMH (r = 0.443, p < 0.01) and DSWMH-F (r = 0.353, p < 0.01). In executive functions, working memory was correlated with PVH (r = -0.266, p < 0.05), flexibility with MTA (r = 0.305, p < 0.01), inhibition performance with PVH (r = 0.280, p < 0.05) and DSWMH (r = 0.282, p < 0.05). Verbal fluency exhibited significant correlations with all of the imaging parameters. An overview of these correlations is shown in Fig. 2. Negative correlations were confirmed with MTA (r = -0.336, p = 0.003; Fig. 2a), PVH (r = -0.391, p < 0.001; Fig. 2b), DSWMH (r = -0.311, p = 0.006; Fig. 2c), and DSWMH-F (r = -0.288, p = 0.013; Fig. 2d). Finally, FAB scores exhibited mild correlations with PVH (r = -0.219, p < 0.05) and DSWMH (r = -0.219, p < 0.05).

**Table 1:** Clinical characteristics of the participants.

Variable	Value
Number (Male / Female)	91 (37 / 54)
Age (years)	75.9 ± 6.3
Education (years)	11.4 ± 3.1
Mini-Mental State Examination	23.5 ± 4.1
Clinical Dementia Rating Scale	0.6 ± 0.3
MTA Z score	2.4 ± 1.1
WMH volume (cm^3)	29.3 ± 23.1
PVH volume (cm^3)	15.3 ± 9.9
DSWMH volume (cm^3)	14.0 ± 15.5
DSWMH-F volume (cm^3)	5.7 ± 6.8

Data are mean ± SD. MTA = medial temporal lobe atrophy; WMH = white matter hyperintensity; PVH = periventricular hyperintensity; DSWMH, deep and subcortical white matter hyperintensity; DSWMH-F = deep and subcortical white matter hyperintensity of frontal lobe.

### Predictive values of MRI variables for cognitive performance

Results from hierarchical multiple linear regression analyses are displayed in Table 3. In accordance with the results of the correlational analysis, MTA was the strongest predictor of flexibility (β = 0.345, p < 0.01) and verbal fluency (β = -0.290, p < 0.01) in executive functions, as well as verbal memory (β = -0.309, p < 0.01), orientation (β = 0.320, p < 0.01), and visuospatial ability (β = -0.310, p < 0.01). Meanwhile, DSWMH was entered as a strong predictor of inhibition performance (β = 0.816, p < 0.01), followed by DSWMH-F (β = 0.623, p < 0.05). Furthermore, PVH was entered as the strongest predictor of FAB score (β = -0.215, p < 0.05), and the second strongest predictor of verbal fluency performance (β = -0.268, p < 0.05).

## DISCUSSION

The current study addressed several issues regarding the relationship between the volume of WMH as well as the Z-score of MTA and various cognitive function tests, including a variety of executive dysfunctions.

**Table 2:** Correlation between cognitive performance and MRI variables.

Cognitive function test (max score)			Value	MTA	PVH	DSWMH	DSWMH-F
**Verbal Memory**		10WDR (10)	2.5 ± 2.4	-0.309^**^	-0.228	-0.028	-0.069
**Orientation**		ADAS-J Orientation	1.5 ± 1.5	0.301^**^	0.155	0.137	0.106
**Visuospatial**		ADAS-J Copy	0.5 ± 0.5	-0.229^**^	0.005	0.225^*^	0.193
**Sustained Attention**		TMT-A	79.7 ± 42.5	-0.074	0.367^**^	0.443^**^	0.353^**^
**Executive functions**	Working Memory	DST-B	3.7 ± 1.0	-0.046	-0.266^*^	-0.172	-0.128
	Inhibition	MST-B	56.8 ± 31.6	0.065	0.280^*^	0.282^*^	0.172
	Flexibility	TMT-B/A	3.4 ± 2.7	0.305^**^	-0.029	-0.142	-0.072
	Verbal Fluency	SVF	11.2 ± 4.4	-0.336^**^	-0.391^**^	-0.311^**^	-0.288^*^
**Screening of Frontal lobe functions**		FAB (18)	13.9 ± 2.7	-0.132	-0.219^*^	-0.219^*^	-0.180

MRI = magnetic resonance image; MTA = medial temporal lobe atrophy; PVH = periventricular hyperintensity; DSWMH = deep and subcortical white matter hyperintensity; DSWMH-F = deep and subcortical white matter hyperintensity of frontal lobe; 10WDR = 10 words delay recall; ADAS-J = Alzheimer Disease Assessment Scale Japanese version; TMT = Trail making test; DST-B = digit span test backward; MST-B = Modified stroop test part B; SVF = semantic verbal fluency; FAB = Frontal assessment battery.

* Significant difference (p < 0.05);

** Significant difference (p < 0.01).

The current results revealed correlations between WMH and the following executive functions: working memory, inhibition, and verbal fluency. Previous studies of WMH suggested that the PVH may be most important for executive functions [1-3]. Our findings are in line with these previous reports, revealing a significant correlation between PVH and these executive functions. However, our regression analysis also indicated that both DSWMH and DSWMH-F were independent predictors of inhibition performance, but not PVH, among the MRI variables. In addition, MTA revealed significant correlations with most of the cognitive function tests that we examined, including verbal memory, orientation, spatial ability, and two executive functions: flexibility and verbal fluency. Overall, the current study revealed a unique association in executive functions between PVH and working memory, DSWMH and inhibition performance, and between MTA and flexibility performance.

**Table 3:** Predictive values of MRI valuables for cognitive performance.

Cognitive function test (max score)			MTA	PVH	DSWMH	DSWMH-F	Age	Education
**Verbal Memory**		10WDR (β)	-0.309^**^	-	-	-	-	-
**Orientation**		ADAS-J Orientation (β)	0.320^**^	-	-	-	-	-0.392^**^
**Visuospatial**		ADAS-J Copy (β)	-0.310^**^	-	-	-	-	-0.230^*^
**Sustained Attention**		TMT-A (β)	-	-	0.405^**^	-	-	-0.379^**^
**Executive functions**	Working Memory	DST-B (β)	-	-	-	-	-	0.258^*^
	Inhibition	MST-B (β)	-	-	0.816^**^	0.623^*^	0.276^*^	-0.287^**^
	Flexibility	TMT-B/A (β)	0.345^**^	-	-	-	-	-
	Verbal Fluency	SVF (β)	-0.290^**^	-0.268^*^	-	-	-	0.409^**^
**Screening of Frontal lobe functions**		FAB (β)	-	-0.215^*^	-	-	-	0.397^**^

MRI = magnetic resonance image; MTA = medial temporal lobe atrophy; PVH = periventricular hyperintensity; DSWMH = deep and subcortical white matter hyperintensity; DSWMH-F = deep and subcortical white matter hyperintensity of frontal lobe; 10WDR = 10 words delay recall; ADAS-J = Alzheimer Disease Assessment Scale Japanese version; TMT = Trail making test; DST-B = digit span test backward; MST-B = Modified stroop test part B; SVF = semantic verbal fluency; FAB = Frontal assessment battery.

* Significant difference (p < 0.05);

** Significant difference (p < 0.01).

The present findings suggest strong biological plausibility. The results of the Rotterdam Scan Study proposed that DSWMH as well as PVH might affect cognitive functions [2]. DSWMH may predominantly disrupt the short association fibers, also known as U or arcuate fibers, which link adjacent gyri. PVH is likely to affect the long association fibers that connect the more distant cortical areas. Decreases in executive cognition are likely to be related to subcortical mechanisms. The connections between the prefrontal cortices and the ascending fiber system consisting of long white matter tracts are considered to play an important role, underlying sustained attention and a variety of executive functions [27]. Our analyses of regions of interest for DSWMH also suggest that the frontal lobe area containing frontal-subcortical circuits reinforce a key role in executive functioning. Recent MRI studies have reported that executive performance is correlated with white matter integrity in the frontal lobe [28, 29]. In contrast, in the current study we did not observe such a correlation for the parieto-occipito-temporal regions of DSWMH.

Interestingly, the flexibility of performance in executive functions was related to MTA, but not WMH. MTA was found to be related to TMT-B in a previous study [30] and a specific role of the temporal lobe in this task has been reported [12]. Although the medial temporal lobe has been traditionally considered to play a role in memory and orientation, the role of MTA should be also considered when examining executive functions. Connections exist between the MTA and prefrontal cortex [11], and there is evidence for a connection between the hippocampus and executive functions [12], which might explain the involvement of MTA in executive functions. Specifically, previous studies point to the importance of a specific hippocampal-prefrontal circuit [11], with regard to executive functions. This implies that the diminished role of this circuit, as a consequence of WMH or MTA, could result in impaired executive functions. The observation of disrupted working memory performance following disconnection of this circuit in rats supports this possibility [31].

Our results are broadly in accord with recent reports of an association between executive dysfunction and lesions in the periventricular and deep subcortical white matter. However, previous studies examining the associations between WMH on MRI and cognitive dysfunction have produced different results [32]. There are several methodological differences between this previous study and the current study. Schmidt et al. (1999) used visual rating scales, whereas we used a semi-automated volumetric method of quantifying WMH. Volumetric WMH measurements are more objective and reliable, and thus provide a more accurate measurement of WMH [19]. In addition, because we distinguished the type of WMH into PVH and DSWMH, it is possible the effect of each volume of WMH on cognitive decline observed in the current study was obscured in the previous study.

A possible limitation of the present study relates to the nature of cross-sectional study designs. Although a number of studies have examined longitudinal cognitive performance in combination with serial MRI measurements [5, 6], most of these found no association between changes in WMH and cognitive functions. It may be valuable to confirm the current findings using longitudinal studies with follow-up MRI and cognitive testing to investigate the association between the progression of WMH, MTA and cognitive decline in elderly subjects. Furthermore, a possible age-associated bias should be considered because several elderly patients over 80 years were included in this study. Another point that warrants caution is that, although several significant associations were noted between WMH, MTA, and cognitive functions, including a variety of executive functions, some of these associations were relatively weak. MRI variables could only account for part of the variation in cognitive performance, implying that other factors, such as age and years of education, were also involved. Finally, normal controls should be included in this study.

Notwithstanding the limitations, the present study provides new insight into the relationship between executive functions and WMH, and how the MTA is involved in this association. In conclusion, the current findings revealed that functional connectivity between the prefrontal cortex and medial temporal lobe play an important role in executive dysfunction in an aging population in the early stages of dementia.

## References

[1] DeCarli C, Murphy DG, Tranh M, Grady CL, Haxby JV, Gillette JA, et al (1995). The effect of white matter hyperintensity volume on brain structure, cognitive performance, and cerebral metabolism of glucose in 51 healthy adults. Neurology, 45: 2077-2084 7501162 10.1212/wnl.45.11.2077

[2] de Groot JC, de Leeuw FE, Oudkerk M, Hofman A, Jolles J, Breteler MM (2001). Cerebral white matter lesions and subjective cognitive dysfunction: the Rotterdam Scan Study. Neurology, 56: 1539-1545 11402112 10.1212/wnl.56.11.1539

[3] Tullberg M, Fletcher E, DeCarli C, Mungas D, Reed BR, Harvey DJ, et al (2004). White matter lesions impair frontal lobe function regardless of their location. Neurology, 63: 246-253 15277616 10.1212/01.wnl.0000130530.55104.b5 PMC1893004

[4] Murray ME, Senjem ML, Petersen RC, Hollman JH, Preboske GM, Weigand SD, et al (2010). Functional impact of white matter hyperintensities in cognitively normal elderly subjects. Arch Neurol, 67: 1379-1385 21060015 10.1001/archneurol.2010.280 PMC3025610

[5] Bolandzadeh N, Davis JC, Tam R, Handy TC, Liu-Ambrose T (2012). The association between cognitive function and white matter lesion location in older adults: a systematic review. BMC Neurol, 12: 126 23110387 10.1186/1471-2377-12-126 PMC3522005

[6] Debette S, Markus HS (2010). The clinical importance of white matter hyperintensities on brain magnetic resonance imaging: systematic review and meta-analysis. Bmj, 341: c3666 20660506 10.1136/bmj.c3666 PMC2910261

[7] Grueter BE, Schulz UG (2012). Age-related cerebral white matter disease (leukoaraiosis): a review. Postgrad Med J, 88: 79-87 22184252 10.1136/postgradmedj-2011-130307

[8] Maniega SM, Valdes Hernandez MC, Clayden JD, Royle NA, Murray C, Morris Z, et al (2015). White matter hyperintensities and normal-appearing white matter integrity in the aging brain. Neurobiol Aging, 36: 909-918 25457555 10.1016/j.neurobiolaging.2014.07.048 PMC4321830

[9] Brickman AM, Sneed JR, Provenzano FA, Garcon E, Johnert L, Muraskin J, et al (2011). Quantitative approaches for assessment of white matter hyperintensities in elderly populations. Psychiatry Res, 193: 101-106 21680159 10.1016/j.pscychresns.2011.03.007 PMC3164869

[10] Smith EE, Salat DH, Jeng J, McCreary CR, Fischl B, Schmahmann JD, et al (2011). Correlations between MRI white matter lesion location and executive function and episodic memory. Neurology, 76: 1492-1499 21518999 10.1212/WNL.0b013e318217e7c8 PMC3087468

[11] Laroche S, Davis S, Jay TM (2000). Plasticity at hippocampal to prefrontal cortex synapses: dual roles in working memory and consolidation. Hippocampus, 10: 438-446 10985283 10.1002/1098-1063(2000)10:4<438::AID-HIPO10>3.0.CO;2-3

[12] Oosterman JM, Vogels RL, van Harten B, Gouw AA, Scheltens P, Poggesi A, et al (2008). The role of white matter hyperintensities and medial temporal lobe atrophy in age-related executive dysfunctioning. Brain Cogn, 68: 128-133 18450353 10.1016/j.bandc.2008.03.006

[13] van den Heuvel DM, ten Dam VH, de Craen AJ, Admiraal-Behloul F, Olofsen H, Bollen EL, et al (2006). Increase in periventricular white matter hyperintensities parallels decline in mental processing speed in a non-demented elderly population. J Neurol Neurosurg Psychiatry, 77: 149-153 16421114 10.1136/jnnp.2005.070193 PMC2077562

[14] Barber R, Gholkar A, Scheltens P, Ballard C, McKeith IG, O'Brien JT (2000). MRI volumetric correlates of white matter lesions in dementia with Lewy bodies and Alzheimer's disease. Int J Geriatr Psychiatry, 15: 911-916 11044873 10.1002/1099-1166(200010)15:10<911::aid-gps217>3.0.co;2-t

[15] Petersen RC, Morris JC (2005). Mild cognitive impairment as a clinical entity and treatment target. Arch Neurol, 62: 1160-1163; discussion 1167 10.1001/archneur.62.7.1160 16009779

[16] McKhann GM, Knopman DS, Chertkow H, Hyman BT, Jack CR Jr., Kawas CH, et al (2011). The diagnosis of dementia due to Alzheimer's disease: recommendations from the National Institute on Aging-Alzheimer's Association workgroups on diagnostic guidelines for Alzheimer's disease. Alzheimers Dement, 7: 263-269 21514250 10.1016/j.jalz.2011.03.005 PMC3312024

[17] Morris JC (1993). The Clinical Dementia Rating (CDR): current version and scoring rules. Neurology, 43: 2412-2414 10.1212/wnl.43.11.2412-a 8232972

[18] Waragai M, Okamura N, Furukawa K, Tashiro M, Furumoto S, Funaki Y, et al (2009). Comparison study of amyloid PET and voxel-based morphometry analysis in mild cognitive impairment and Alzheimer's disease. J Neurol Sci, 285: 100-108 19552926 10.1016/j.jns.2009.06.005

[19] Hirata Y, Matsuda H, Nemoto K, Ohnishi T, Hirao K, Yamashita F, et al (2005). Voxel-based morphometry to discriminate early Alzheimer's disease from controls. Neurosci Lett, 382: 269-274 15925102 10.1016/j.neulet.2005.03.038

[20] Folstein MF, Folstein SE, McHugh PR (1975). "Mini-mental state". A practical method for grading the cognitive state of patients for the clinician. J Psychiatr Res, 12: 189-198 1202204 10.1016/0022-3956(75)90026-6

[21] Ralph MR (1958). Validity of the Trail Making Test as an Indicator of Organic Brain Damage. Perceptual and Motor Skills, 8: 271-276

[22] Alan SK (1983). Test Review: Wechsler, D. Manual for the Wechsler Adult Intelligence Scale, Revised. New York: Psychological Corporation, 1981. Journal of Psychoeducational Assessment, 1: 309-313

[23] Kimble MO, Frueh BC, Marks L (2009). Does the modified Stroop effect exist in PTSD? Evidence from dissertation abstracts and the peer reviewed literature. J Anxiety Disord, 23: 650-655 19272751 10.1016/j.janxdis.2009.02.002 PMC2844871

[24] Zarino B, Crespi M, Launi M, Casarotti A (2014). A new standardization of semantic verbal fluency test. Neurol Sci, 35: 1405-1411 24705901 10.1007/s10072-014-1729-1

[25] Dubois B, Slachevsky A, Litvan I, Pillon B (2000). The FAB: a Frontal Assessment Battery at bedside. Neurology, 55: 1621-1626 11113214 10.1212/wnl.55.11.1621

[26] Sano M, Raman R, Emond J, Thomas RG, Petersen R, Schneider LS, et al (2011). Adding delayed recall to the Alzheimer Disease Assessment Scale is useful in studies of mild cognitive impairment but not Alzheimer disease. Alzheimer Dis Assoc Disord, 25: 122-127 20921876 10.1097/WAD.0b013e3181f883b7 PMC3526369

[27] Tisserand DJ, Jolles J (2003). On the involvement of prefrontal networks in cognitive ageing. Cortex, 39: 1107-1128 14584569 10.1016/s0010-9452(08)70880-3

[28] Kochunov P, Coyle T, Lancaster J, Robin DA, Hardies J, Kochunov V, et al (2010). Processing speed is correlated with cerebral health markers in the frontal lobes as quantified by neuroimaging. NeuroImage, 49: 1190-1199 19796691 10.1016/j.neuroimage.2009.09.052 PMC2789896

[29] Bartzokis G, Lu PH, Tingus K, Mendez MF, Richard A, Peters DG, et al (2010). Lifespan trajectory of myelin integrity and maximum motor speed. Neurobiology of Aging, 31: 1554-1562 18926601 10.1016/j.neurobiolaging.2008.08.015 PMC2888859

[30] van der Flier WM, Middelkoop HA, Weverling-Rijnsburger AW, Admiraal-Behloul F, Bollen EL, Westendorp RG, et al (2005). Neuropsychological correlates of MRI measures in the continuum of cognitive decline at old age. Dement Geriatr Cogn Disord, 20: 82-88 15942197 10.1159/000086072

[31] Wang GW, Cai JX (2006). Disconnection of the hippocampal-prefrontal cortical circuits impairs spatial working memory performance in rats. Behav Brain Res, 175: 329-336 17045348 10.1016/j.bbr.2006.09.002

[32] Schmidt R, Fazekas F, Kapeller P, Schmidt H, Hartung HP (1999). MRI white matter hyperintensities: three-year follow-up of the Austrian Stroke Prevention Study. Neurology, 53: 132-139 10408549 10.1212/wnl.53.1.132

